# β-glucan Exposure on the Fungal Cell Wall Tightly Correlates with Competitive Fitness of *Candida* Species in the Mouse Gastrointestinal Tract

**DOI:** 10.3389/fcimb.2016.00186

**Published:** 2016-12-22

**Authors:** XiaoHui Sem, Giang T. T. Le, Alrina S. M. Tan, Gloria Tso, Marina Yurieva, Webber W. P. Liao, Josephine Lum, Kandhadayar G. Srinivasan, Michael Poidinger, Francesca Zolezzi, Norman Pavelka

**Affiliations:** Singapore Immunology Network, Agency for Science, Technology and ResearchSingapore, Singapore

**Keywords:** *Candida*, *Candida albicans*, cell wall, competitive fitness, gastrointestinal tract, beta-glucan, dectin-1

## Abstract

*Candida albicans* is responsible for ~400,000 systemic fungal infections annually, with an associated mortality rate of 46–75%. The human gastrointestinal (GI) tract represents the largest natural reservoir of *Candida* species and is a major source of systemic fungal infections. However, the factors that control GI colonization by *Candida* species are not completely understood. We hypothesized that the fungal cell wall would play an important role in determining the competitive fitness of *Candida* species in the mammalian GI tract. To test this hypothesis, we generated a systematic collection of isogenic *C. albicans* cell wall mutants and measured their fitness in the mouse GI tract via quantitative competition assays. Whereas a large variation in competitive fitness was found among mutants, no correlation was observed between GI fitness and total levels of individual cell wall components. Similar results were obtained in a set of distantly-related *Candida* species, suggesting that total amounts of individual cell wall components do not determine the ability of fungi to colonize the GI tract. We then subjected this collection of *Candida* strains and species to an extensive quantitative phenotypic profiling in search for features that might be responsible for their differences in GI fitness, but found no association with the ability to grow in GI-mimicking and stressful environments or with *in vitro* and *in vivo* virulence. The most significant association with GI fitness was found to be the strength of signaling through the Dectin-1 receptor. Using a quantitative assay to measure the amount of exposed β-glucan on the surface of fungal cells, we found this parameter, unlike total β-glucan levels, to be strongly predictive of competitive fitness in the mouse GI tract. These data suggest that fungal cell wall architecture, more so than its crude composition, critically determines the ability of fungi to colonize the mammalian GI tract. In particular, recognition of exposed β-glucan by Dectin-1 receptor appears to severely limit *Candida* GI fitness and hence represents a promising target to reduce fungal colonization in patients at risks of systemic candidiasis.

## Introduction

The mammalian gastrointestinal (GI) tract is a complex environment hosting a large number and variety of microbes that include not only bacteria but also fungi (Qin et al., 2010; Iliev et al., 2012). While much effort has been placed in unraveling microbiota associations with host health and disease, little is known about factors that determine the ability of individual microbes, and especially fungi, to colonize the GI environment. The most frequently isolated fungi from the human GI tract belong to the *Candida* genus, with *C. albicans* consistently ranking as the most successful fungal colonizer in industrialized countries, followed by *C. tropicalis, C. glabrata, C. krusei, C. parapsilosis*, and *C. dubliniensis* (Maccallum, 2010). In addition to acting as commensals in asymptomatic individuals, *Candida* species are also the most important opportunistic fungal pathogens of humans (Brown et al., 2012). *Candida* species are able to infect the skin, mucosae and in some cases even the bloodstream of patients, especially those with a weakened immunity (Pfaller and Diekema, 2007). Clinical studies have shown that a large fraction of life-threatening systemic infections by *C. albicans* originate from strains residing in the patients' own GI tract (Nucci and Anaissie, 2001; Miranda et al., 2009). Therefore, understanding the factors that control the ability of *Candida* species to colonize the mammalian GI tract might open up opportunities for novel prevention strategies of systemic candidiasis.

Innate immunity plays a paramount role in controlling fungal infections and in limiting the systemic dissemination of *Candida* species from the GI tract (Koh et al., 2008). The *Candida* cell wall is an intricate matrix of molecular interactions between pathogen-associated molecular patterns (PAMPs) on the fungal surface and pathogen-recognition receptors (PRRs) on innate immune cells (Netea et al., 2008). These PAMP-PRR interactions are crucial not only for the recognition of fungal pathogens, but also for the initiation of appropriate immune responses (Gow and Hube, 2012). Whereas the role of the *Candida* cell wall composition has been extensively studied during *in vitro* interactions with innate immune cells and during *in vivo* systemic infections, little is known about its role during asymptomatic colonization of the mammalian GI tract.

**Figure 1:**
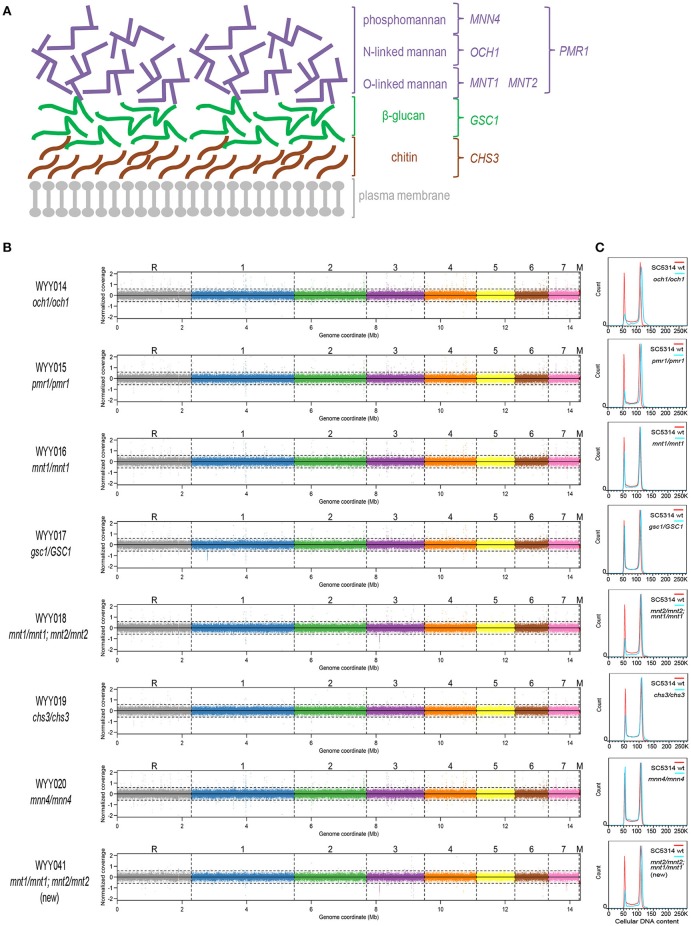
**Euploidy and isogenicity of *C. albicans* cell wall mutants. (A)** Schematic representation of the major cell wall components of *C. albicans*. The mannan (phosphomannan, N-linked mannans and O-linked mannans, in purple), β-glucan (in green) and chitin layers (in brown) are located externally to the plasma membrane (in gray). In this study, several genes involved in modifying and regulating each cell wall component were perturbed and investigated. They are listed in colors corresponding to each of the three layers. **(B)** The aforementioned cell wall mutants were subjected to deep sequencing and compared to the parental strain. The coverage plots, with a different color for each chromosome, illustrate lack of segmental or whole-chromosome aneuploidy (M, mitochondrial DNA). Apart from a few single-nucleotide substitutions, all sequenced mutants were perfectly isogenic to the wild-type *C. albicans* parental strain SC5314. **(C)** Cell wall mutants (blue) generated in the parental SC5314 background were analyzed for their cellular DNA content by flow cytometry and compared to that of the parental strain (red).

The fungal cell wall is organized into a complex network of polysaccharides that are organized in layers (Figure 1A), each eliciting distinct immune responses. The innermost cell wall layer, just above the plasma membrane, is composed of chitin. It is primarily synthesized by chitin synthase 3 (encoded by *CHS3*) and blocks pro-inflammatory cytokine production (Bulawa et al., 1995; Mora-Montes et al., 2011). The intermediate β-glucan layer is critically dependent on (1,3)-β-glucan synthase activity (whose essential catalytic subunit is encoded by *GSC1*) and triggers instead strong inflammatory responses (Mio et al., 1997; Brown et al., 2003; Gow et al., 2007). This β-glucan layer is however normally masked by the less pro-inflammatory outermost mannan layer (critically dependent on *PMR1* for its synthesis), which includes O-linked mannans (dependent on *MNT1* and *MNT2*), N-linked mannans (dependent on *OCH1*) and phosphomannans (dependent on *MNN4*). This relatively ordered structure is however far from static, as the intermediate β-glucan layer can be exposed or change its conformation during infection and morphogenesis (Wheeler et al., 2008; Lowman et al., 2014). Moreover, the same cell wall layer can mediate different outcomes in different pathophysiological contexts. While Dectin-1-mediated recognition of β-glucan is for instance required for controlling systemic infections (Taylor et al., 2007) and even triggers a protective “training” of innate immunity (Quintin et al., 2012), it appears to be dispensable for controlling asymptomatic GI colonization (Vautier et al., 2012). However, the latter findings were obtained by employing a wild-type *C. albicans* strain, which is known to mask its β-glucan layer relatively well, hence it is currently unknown whether variation in β-glucan exposure across *Candida* strains and species underlies their variable ability to colonize the mammalian GI tract.

Here we employed a reverse genetic approach coupled with an extensive quantitative phenotypic characterization to systematically perturb the main polysaccharide layers of the *C. albicans* cell wall and assess their contribution in a mouse model of GI colonization. We also took advantage of the natural variation of cell wall architectures that exists within the *Candida* genus to validate our findings. Our data indicate that whereas most genetic perturbations in the *C. albicans* cell wall reduce GI fitness, several *Candida* species are actually fitter than *C. albicans* in this environment. Interestingly, the strength of signaling through the Dectin-1 receptor emerged as the strongest predictor of GI fitness across all analyzed strains and species, while no correlation was found with crude levels of any tested cell wall component. As predicted, *Candida* strains and species exhibiting the strongest signaling through the Dectin-1 receptor also exposed the highest amounts of β-glucan on their cell surface. Overall, these findings demonstrate a fundamental role of the *Candida* cell wall architecture in determining fungal colonization of the mammalian GI tract and open up new avenues for the prevention and control of opportunistic fungal infections.

## Materials and methods

### Fungal strains

*C. albicans* strains and other *Candida* species used in this study are summarized in Supplementary Table 1. Details about culture conditions and strain construction, including primers used in this study (Supplementary Table 2), can be found in Supplemental Methods. The ploidy of all generated strains was determined via a previously described quantitative flow cytometric method (Pavelka et al., 2010).

### Whole-genome sequencing

Genomic DNA was extracted as described (Rancati et al., 2008). Sequencing libraries were prepared using Illumina sample preparation kits and indexed paired-end (PE) sequences were obtained on an Illumina HiSeq platform. All sequencing data were deposited in NCBI SRA database under accession number SRP056269. Data pre-processing, mapping and variant calling was performed in CLC Genomics Workbench, essentially as described (Liu et al., 2015). Higher level data integration and analysis was performed via customized R scripts. Detailed sequencing protocols and analysis parameters are provided in Supplemental Methods. A summary of the sequencing data and results is reported in Supplementary Tables 3, 4.

### Cell wall composition analysis

Logarithmically-growing *C. albicans* was washed three times in 50 ml of 1 mM cold phenylmethanesulfonyl fluoride (PMSF). 3 volumes of glass beads were then added to one volume of pellet and vortexed for 10–15 times at 1-min intervals to break the cells. The cell lysate was again washed three times with 50 ml cold PMSF, before addition of 3 volumes of 2% SDS to the pellet and incubation for 10 min at 100°C for two rounds. SDS was removed by washing the pellet in 1 mM PMSF and the pellet was dried overnight at 55°C before re-suspension in 1 mM PMSF to a solution of 0.01 g dry weight / ml and storage at −20°C. Total chitin was extracted and measured as described (Kapteyn et al., 1997). Total mannan were extracted from isolated cell walls by alkali treatment and further precipitated with Fehling's solution as a copper complex as described (Chaffin et al., 1998). Total glucan was extracted as described (Dijkgraaf et al., 1996) and the extracted glucan and mannan components were then measured by the Dubois method (Dubois et al., 1956) using glucose as a standard.

### Mouse experiments

Mouse experiments were conducted according to the rules and guidelines of the Agri-Food and Veterinary Authority (AVA) and the National Advisory Committee for Laboratory Animal Research (NACLAR), Singapore. The experiments were reviewed and approved by the Institutional Review Board of the Biological Resource Center, Singapore (IACUC protocol 140955). Six to ten weeks-old female C57BL/6 wild-type mice were used for all experiments. Systemic virulence was assessed after injection of 5 × 10^5^ CFUs via the tail vein. GI competition experiments were performed essentially as described in Prieto et al. (2014). Briefly, equimolar mixtures of a test strain and a fluorescently tagged (dTomato-expressing) reference strain were intragastrically inoculated in antibiotic-treated mice (2 mg/ml streptomycin + 1500 U/ml penicillin G) and their relative frequency was monitored over time by plating stool homogenates on antibiotics-containing plates and counting fluorescent colonies under a fluorescence stereomicroscope. The relative fitness coefficient of the test strain was then obtained by linear regression according to the following formula:

(1) $$log_{2}[R(t)/R(t_{0})]=s\gamma_{R}(t-t_{0}),$$

where *R*(*i*) represents the ratio between the test strain and the reference strain at time *i*; *s* is the selection coefficient; γ_*R*_ is the growth rate of the reference strain expressed as cell divisions per hour; *t* represents the time points in hours and *t*_0_ the initial time point. *In vitro* fitness competition assays were performed essentially in the same way, except that strain mixtures were serially passaged on a daily basis in YPD medium. More details are provided in the Supplemental Methods.

### *Candida*-macrophage co-cultures

J774.1 cells were plated at a density of 5 × 10^5^ cells/well in 12-well plates for 24 h. *C. albicans* strains from overnight cultures were washed twice in PBS and co-cultured with the macrophages at various MOIs, ranging from 1:200 to 1:1600. Under these conditions, >99% of individual *C. albicans* cells were phagocytosed by the macrophages after 3 h (data not shown). Visible fungal colonies were counted after 24 h, and the percentage of phagosome-escaping cells was determined by linear regression as explained in the Supplemental Methods.

### Quantitative resistance assays

Resistance to mCRAMP was determined on logarithmically growing cells re-suspended in sterile PBS and incubated at 37°C for 1–2 h. Cell pellets were then re-suspended in a 2-fold serial dilution of mCRAMP 1–39 (Synpeptide) and incubated at 37°C for 2 h, followed by a PBS wash, live/dead staining with 30 μM propidium iodide for 15 min and flow cytometric analysis. Half-maximal effective concentrations (EC_50_) were determined by non-linear regression in GraphPad Prism. The half-maximal inhibitory concentrations (IC_50_) of acetic and lactic acid on fungal growth were determined *in vitro* via turbidimetric assays as described previously (Cottier et al., 2015a) with some minor modifications (refer to Supplemental Methods). Oxidative stress resistance was determined on PBS-washed overnight cultures incubated in PBS at 37°C for 2 h. Cells were then treated with a range of *tert*-Butyl hydroperoxide (*t*BOOH) concentrations, incubated at 37°C for 1 h and subjected to live/dead analysis as described above. The NO sensitivity assay was performed following previous protocol (Chiranand et al., 2008) with some minor modifications (refer to Supplemental Methods).

### High-throughput phenotypic profiling

Overnight cultures of freshly revitalized *C. albicans* strains and *Candida* spp. were normalized to a final OD_600_ of 2 and washed once with PBS. A Freedom EVO 150 liquid-handling robot (Tecan) performed serial dilutions of the cultures and spotted 3 μl of each serial dilution onto YPD agar omnitrays representing one of a wide range of growth conditions listed in Supplementary Table 5. Each condition was tested a minimum of three independent times and growth data was acquired by a desktop scanner and analyzed using a custom R script for automated spot detection and intensity measurements followed by non-linear curve fitting across the range of serial dilutions and determination of relative growth scores as explained in Supplemental Methods.

### Quantification of β-1, 3-glucan exposure

Logarithmically-growing *Candida* cells were stained with anti-β-1,3-glucan monoclonal antibody (mouse IgG; Biosupplies) followed by anti-mouse IgG Alexa Fluor 488 (Molecular Probes) staining, as described previously (Wartenberg et al., 2014) with some modifications (refer to Supplemental Methods). The level of β-1,3-glucan exposure on the cell surface was then quantified on a MACSquant VYB flow cytometer (Miltenyi).

### Quantification of PRR signaling strength

*Dectin-1, Dectin-2, TLR2*, and *TLR4* signaling measurements were performed using a NF-kB reporter cell line (HEK-Blue™ hDectin-1b, HEK-Blue™ mDectin-2 Cells, HEK-Blue™ mTLR2 cells, HEK-Blue™ mTLR4 cells Invivogen) and its parental cell line (HEK-Blue™-null1-v or HEK-Blue™ null2 cells) as the negative control. Briefly, 1 × 10^5^ cells/well were stimulated with 1 × 10^6^ formaldehyde-fixed *Candida* cells/well and incubated in 96-well flat bottom plates for 6-20 h. Finally, NF-kB activity was quantified with the QUANTI-Blue (Invivogen) reagent accordingly to the manufacturer's instructions.

### Statistical analysis

All data were analyzed using Excel (Microsoft) and Prism (Graphpad) software. Data is reported as mean ± SD. To compare wild-type *C. albicans*, isogenic *C. albicans* cell wall mutants and *Candida* spp. strains fitter than or as fit as wild-type *C. albicans* and isogenic *C. albicans* cell wall mutants and *Candida* spp. strains less fit than wild-type *C. albicans*, non-parametric Mann-Whitney test was used. For all other comparisons, statistical significance was calculated using two-tailed unpaired *t*-tests with Welch's correction. In all analyses, *p* ≤ 0.05 were regarded as statistically significant.

## Results

### A collection of isogenic *C. albicans* cell wall mutants

To assess the contribution of each cell wall component to GI tract fitness, we first generated an isogenic set of *C. albicans* mutants in the prototroph and otherwise wild-type reference strain SC5314, each deleted in one or more genes illustrated in Figure 1A. In particular, we generated homozygous mutants in *CHS3, PMR1, MNT1, OCH1*, and *MNN4* using the SAT-Flipper technique, which allows inducible removal of the deletion cassette, leaving no ectopic selection marker in the genome (Sasse and Morschhauser, 2012). Since *GSC1* is an essential gene, we successfully generated the corresponding heterozygous but not the homozygous mutant; on the other hand, because *MNT1* was shown to be partially redundant with *MNT2* (Munro et al., 2005), we also generated a double homozygous strain carrying both gene deletions in homozygosity. Using quantitative staining of cellular DNA content followed by flow cytometry, we confirmed all strains in the collection to be diploid (Figure 1B). Whole-genome sequencing further indicated absence of either whole-chromosome or segmental aneuploidy and presence of only a few point mutations, most of which were heterozygous and either non-coding or synonymous (Figure 1C, Supplementary Table 4). The *mnt1*/*mnt1*;*mnt2*/*mnt2* strain was the only strain containing a non-synonymous mutation in homozygosity and was therefore reconstructed. The reconstructed *mnt1*/*mnt1*;*mnt2*/*mnt2* strain was then re-sequenced and found not to carry this or other homozygous non-synonymous mutation (Supplementary Table 4). Experiments critical for this study have been repeated in parallel using both *mnt1*/*mnt1*;*mnt2*/*mnt2* strains and found not to lead to significantly different outcomes (Supplementary Figure 1). This collection of *C. albicans* cell wall mutants therefore represents a carefully constructed isogenic set of strains that is well suited for quantitative phenotypic analyses without potential confounders due to undocumented genetic or karyotypic differences. Moreover, since these strains were generated without using any auxotrophic marker, they are expected to show no intrinsic deficiency in their ability to grow in nutrient-limited environments, e.g., during colonization or infection of certain host tissues, and should therefore represent an invaluable resource to study the role of the *C. albicans* cell wall during interaction with the host.

### GI tract fitness of *C. albicans* cell wall mutants

**Figure 2:**
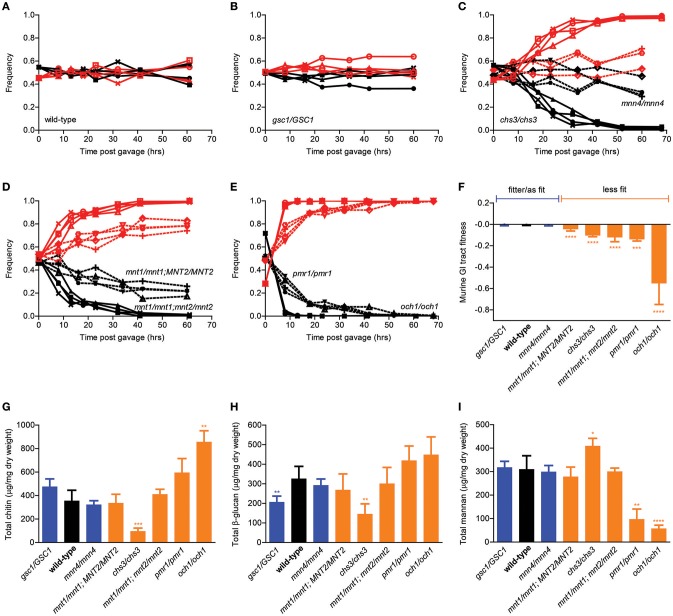
**Murine GI tract fitness of isogenic *C. albicans* cell wall mutants. (A–E)** Relative frequency of wild-type *C. albicans* (**A**, black line) and the isogenic cell wall mutants *gsc1/GSC1* (**B**, black lines), *mnn4/mnn4* (**C**, black dotted lines), *chs3/chs3*, (**C**, black solid lines), *mnt1/mnt1; MNT2/MNT2* (**D**, black dotted lines), *mnt1/mnt1; mnt2/mnt2* (**D**, black solid lines), *pmr1/pmr1* (**E**, black dotted lines) and *och1/och1* (**E**, black solid lines) in comparison to SC5314-dTomato (**A–E**, red lines) during an *in vivo* competition assay in the mouse GI tract. All experiments were initiated at a nominal ratio of 1:1 between competing strains, except for *och1/och1* where the *och1/och1*:SC5314-dTomato ratio was 7:3. Each pair of lines represents an independent biological assay with a singly-housed mouse, with ≥4 mice per strain and ≥200 CFUs counted per time point. **(F)** Murine GI tract fitness of wild-type *C. albicans* (black) and the isogenic cell wall mutants are summarized and represented as average and standard deviation of fitted selection coefficients (see Supplemental Methods). Wild-type and mutant strains are ranked in decreasing order of murine GI tract fitness and classified into two groups: fitter than or as fit as wild-type (blue); less fit than wild-type (orange). **(G–I)** Cell wall polysaccharide composition of each wild-type and mutant strain in terms of total chitin **(G)**, total β-glucan **(H)** and total mannan **(I)**. Each bar graph represents the average of 3 independent biological assays. Strains are ranked and classified as in **(F)**. Asterisks shown represent significant two-tailed *p*-values obtained from unpaired Welch's *t*-tests between wild-type and mutant strain (^*^ < 0.05; ^**^ < 0.01; ^***^ < 0.001; ^****^ < 0.0001).

We next adopted a previously proposed *in vivo* competition assay aimed at comparing the ability of different *C. albicans* strains to colonize the mouse GI tract (Prieto et al., 2014) and extended it into a quantitative *in vivo* competitive fitness assay. Briefly, the original method entailed orally gavaging antibiotic-treated mice with equimolar mixtures of a test strain and a fluorescently tagged reference strain, followed by counting fluorescent and non-fluorescent colonies in plated serial dilutions of stool samples collected at specific time points from the double-colonized mice. As expected, competition of wild-type SC5314 cells against the fluorescently tagged version of the SC5314 strain led to no significant change in the relative proportion of fluorescent colonies in the mouse stool over time, indicating that presence of the fluorescence cassette was associated with no or minimal fitness cost in the mouse GI tract (Figure 2A). In contrast, our collection of isogenic cell wall mutants displayed a variety of behaviors in this assay, ranging from undetectable to significant decrease in relative frequency depending on the genotype (Figures 2B–E). In particular, while the *gsc1*/*GSC1* and the *mnn4*/*mnn4* mutants were essentially undistinguishable from the wild-type strain, the *mnt1*/*mnt1* strain showed a consistent and progressive drop in relative frequency during the competition assay, reaching ~20% after ~60 h. The *mnt1*/*mnt1*; *mnt2*/*mnt2* mutant, however, was completely outcompeted by the fluorescent strain in <40 h, suggesting that *MNT1* and *MNT2* are not completely redundant with each other for GI tract fitness. Moreover, while *chs3*/*chs3* and *pmr1*/*pmr1* mutants were outcompeted by the reference strain in a similar amount of time as the *mnt1*/*mnt1*;*mnt2*/*mnt2* strain, the *och1*/*och1* strain was outcompeted in ~10 h.

We then developed a regression method that allows us to rigorously quantitate the relative competitive fitness of each test strain using a time series of relative frequencies of fluorescent colonies as an input (see Supplemental Methods for details). By this analysis, the *gsc1*/*GSC1* and *mnn4*/*mnn4* strains were at least as fit as wild-type *C. albicans*, while all other cell wall mutants in this study displayed a wide range of fitness disadvantages (Figure 2F). In an attempt to correlate GI tract fitness with cell wall composition, we then quantified the total amounts of chitin, β-glucans and mannans in all strains but no significant association was found. In particular, among the strains with significantly reduced GI tract fitness, *mnt*/*mnt1;MNT2/MNT2* and *mnt1*/*mnt1*;*mnt2*/*mnt2* showed no significant alterations in crude cell wall composition; on the other hand, *chs3*/*chs3* had significantly reduced chitin and β-glucan and significantly increased mannan levels and *och1*/*och1* had significantly increased chitin and significantly decreased mannan levels. Overall, while genetic alterations in cell wall biosynthetic pathways clearly affect the ability of *C. albicans* to colonize the mouse GI tract, differential fitness in this environment cannot be attributed to relative compositional differences in the three main polysaccharide layers.

### GI tract fitness of *Candida* species

**Figure 3:**
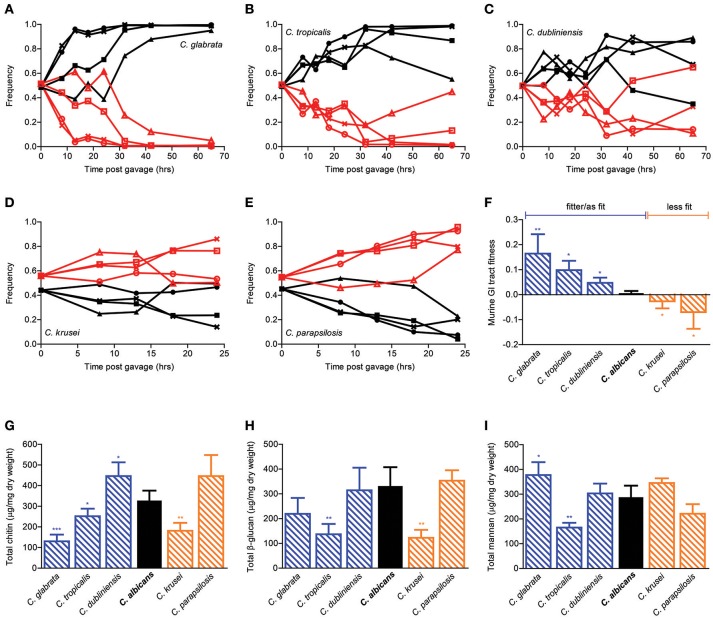
**Murine GI tract fitness of *Candida* spp. strains**. Relative frequency of wild-type *C. glabrata* (**A**, black), *C. tropicalis* (**B**, black), *C. dubliniensis* (**C**, black), *C. krusei*, (**D**, black), and *C. parapsilosis* (**E**, black) in comparison to SC5314-dTomato (**A–E**, red) during a competition assay in the mouse GI tract, represented as in Figures 2A–E. Each pair of lines represents an independent biological assay with a singly-housed mouse, with ≥4 mice per strain and ≥200 CFUs counted per time point. **(F)** Murine GI tract fitness of *C. albicans* (black) and *Candida* spp. (blue and orange shaded bars) represented as in Figure 2F. **(G–I)** Cell wall polysaccharide composition of each *Candida* species represented as in Figures 2G–I. Asterisks shown represent significant two-tailed *p*-values obtained from unpaired Welch's *t*-tests between wild-type and mutant strain (^*^ < 0.05; ^**^ < 0.01; ^***^ < 0.001; ^****^ < 0.0001).

In order to extend the generality of our findings beyond *C. albicans*, we then repeated the *in vivo* competition assays in distantly related *Candida* species (Figures 3A–E). Interestingly, while *C. krusei* and *C. parapsilosis* were significantly less fit, *C. glabrata, C. tropicalis*, and *C. dubliniensis* were significantly fitter than *C. albicans* in the mouse GI tract (Figure 3F). In accordance with the cell wall mutants' data, compositional analysis of the major cell wall polysaccharides did not yield any significant association with GI tract fitness. In particular, whereas both *C. glabrata* and *C. dubliniensis* were significantly fitter than *C. albicans* in the mouse GI environment, the former had significantly reduced and the latter significantly increased chitin levels in comparison to *C. albicans*. Moreover, whereas both *C. tropicalis* and *C. krusei* showed significantly decreased β-glucan levels, the former was significantly fitter and the latter significantly less fit then *C. albicans*. Hence, despite the existence of a large variation in both GI tract fitness and cell wall composition within the *Candida* genus, these two traits appear to be largely independent from each other.

### Quantitative phenotypic profiling reveals no associations with GI tract fitness

**Figure 4:**
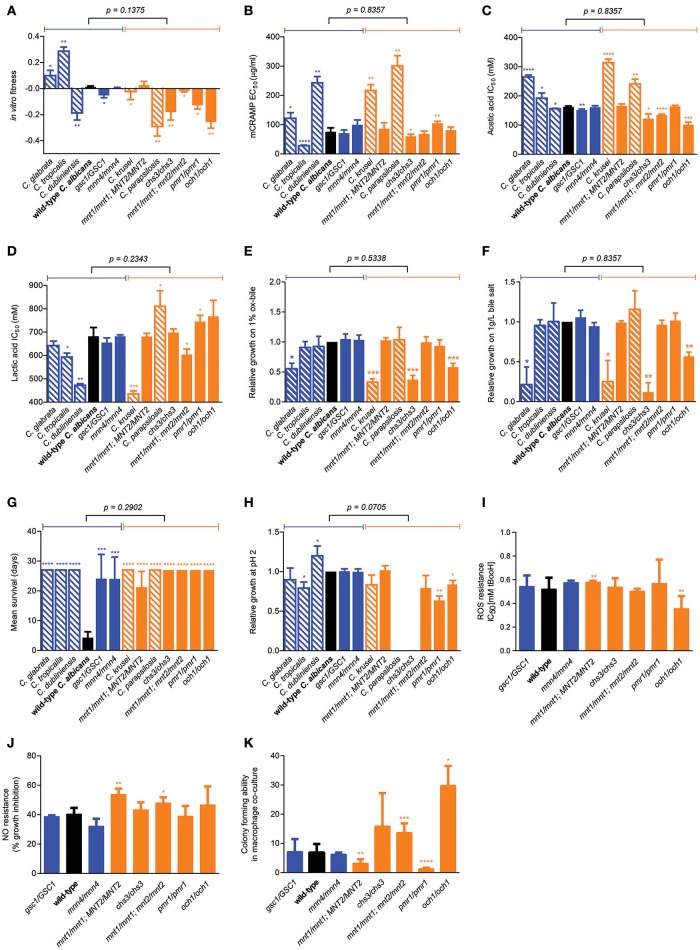
**Factors not associated with murine GI tract fitness. (A)** Wild-type *C. albicans* (black) and the isogenic cell wall mutants (blue and orange) were assessed for their *in vitro* fitness in YPD. **(B–K)** Using various experimental procedures, all wild-type *C. albicans* (black bars), isogenic *C. albicans* cell wall mutants (blue and orange solid bars) and *Candida spp*. strains (blue and orange shaded bars) were assessed for their resistance to acute exposure to **(B)** mCRAMP, **(C)** acetic acid, **(D)** lactic acid, **(E)** Ox-bile, and **(F)** bile-salt and for their **(G)** systemic virulence in wild-type C57BL/6 mice, **(H)** relative growth at pH 2, **(I)** resistance to ROS, **(J)** resistance to NO and **(K)** their survivability (colony-forming ability) after 24 h co-culture with the J774A.1 murine macrophage cell line. Strains are ranked in decreasing order of murine GI tract fitness as in Supplementary Figure 2A, and classified into two groups: fitter than or as fit as wild-type *C. albicans* (solid or shaded blue); less fit than wild-type *C. albicans* (solid or shaded orange). The *p*-value on top of each panel was obtained from a Mann-Whitney test between the two indicated groups. Asterisks represent significant two-tailed *p*-values from unpaired Welch's *t*-tests in comparison to wild-type *C. albicans* (^*^ < 0.05; ^**^ < 0.01; ^***^ < 0.001; ^****^ < 0.0001). Each measurement was obtained from ≥3 independent biological experiments.

We next sought to identify factors underlying the variation in mouse GI tract fitness across all our *C. albicans* cell wall mutants and the other *Candida* species, employing a systematic profiling of a large number of quantitative phenotypic parameters (Supplementary Figure 2). Consistent with above findings, no significant association was found between competitive fitness in the mouse GI tract (Supplementary Figure 3A) and total chitin, mannan, β-glucan, β-1,6-glucan or β-1,3-glucan levels (Supplementary Figures 3B–F). Moreover, no association was seen between *in vivo* and *in vitro* fitness (Figure 4A), or between *in vivo* fitness and ability to grow under a variety of physiological or stressful environmental conditions (Supplementary Figures 3G–R), ruling out simple explanations based on overall fitness effects in some of the tested strains. Secretion of the mouse cathelicidin-related antimicrobial peptide (mCRAMP) in the GI lumen was recently shown to play an important role in controlling *C. albicans* colonization in the mouse GI tract (Fan et al., 2015); however no significant correlation between mouse GI tract fitness and mCRAMP resistance was observed (Figure 4B). Microbiota-derived weak organic acids, such as acetic and lactic acid, were shown to be fungistatic and proposed to contribute to colonization resistance against *C. albicans* in the mouse GI tract (Cottier et al., 2015b), however *Candida* strains and species displaying the highest competitive fitness in the mouse GI tract did not show a significant increase in acetic acid or lactic acid resistance (Figures 4C,D). An important feature of gut commensal microbes is their ability to survive in the presence of bile (Strati et al., 2016), but GI fitness was uncorrelated to resistance to ox bile or bile salts (Figures 4E,F). A trade-off between gut commensalism and systemic virulence was recently proposed (Pande et al., 2013), but no association was found between GI tract fitness and ability to kill mice during systemic infections, as all tested cell wall mutants and *Candida* species were significantly less virulent that wild-type *C. albicans* (Figure 4G). Moreover, with the only exception of the *och1* mutant, the reduced systemic virulence exhibited by the *C. albicans* cell wall mutants did not associate with the ability to survive in phagosome-like conditions (low pH, high ROS, high NO) (Figures 4H–J). Indeed, none of the cell wall mutants displayed obvious hyphal morphogenesis defects (Supplementary Figure 4) and their ability to escape from macrophages after phagocytosis associated neither with systemic virulence nor with GI tract fitness (Figure 4K).

### Role of β-glucan/dectin-1 signaling axis in determining fungal GI colonization

**Figure 5:**
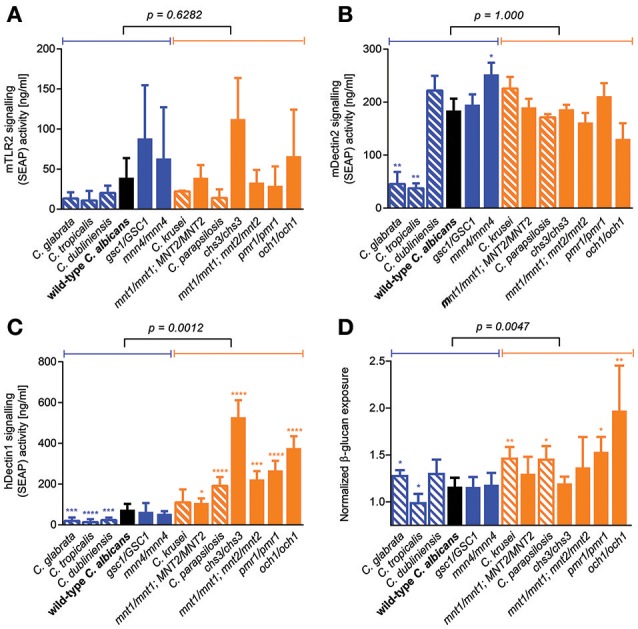
**Role of β-glucan/Dectin-1 signaling axis in determining murine GI tract fitness. (A–D)** Wild-type *C. albicans* (black), isogenic *C. albicans* cell wall mutants (blue and orange solid bars) and *Candida* spp. strains (blue and orange shaded bars) were assessed for their ability to activate TLR-2 receptor signaling in **(A)** HEK-Blue mTLR-2 cells, Dectin-2 receptor signaling in **(B)** HEK-Blue mDectin-2 cells, Dectin-1 receptor signaling in **(C)** HEK-Blue hDectin-1b cells and β-glucan exposure on their cell walls using an anti-β-glucan monoclonal antibody **(D)**. Wild-type and mutant strains are ranked in decreasing order of murine GI tract fitness and classified into two groups: fitter than or as fit as wild-type *C. albicans* (solid or shaded blue); less fit than wild-type *C. albicans* (solid or shaded orange). The *p*-value on top of each panel was obtained from a Mann-Whitney test between the two indicated groups. Asterisks represent significant two-tailed *p*-values from unpaired Welch's *t*-tests in comparison to wild-type *C. albicans* (^*^ < 0.05; ^**^ < 0.01; ^***^ < 0.001; ^****^ < 0.0001). Each measurement was obtained from ≥3 independent biological experiments.

Because GI tract fitness was not associated with any plausible microbe-intrinsic factor or phenotype that we tested so far, we reasoned that the ability of fungal cells to colonize the gut environment might be better explained by factors related to host-microbe interactions. We therefore screened our library of *C. albicans* cell wall mutants and *Candida* species for their ability to trigger signal transduction events downstream of some of the main PRRs known to be important for fungal cell recognition. To this end, we employed commercially available human cell lines expressing individual PRRs and an inducible NF-κB reporter. While TLR4 signaling was essentially undetectable in response to any tested strain (data not shown), large variation in the ability to trigger TLR2- and Dectin-2-mediated signaling was observed between the *Candida* strains and species; this however did not associate with differences in GI tract fitness (Figures 5A,B). Interestingly, *Candida* strains and species displayed a wide range of abilities to signal through the Dectin-1 receptor, and this was significantly associated with their competitive fitness in the mouse GI tract (Figure 5C). This prompted us to reevaluate our initial observation about the lack of association between GI fitness and cell wall composition, and to investigate a potential contribution not of the total amount but of the level of exposure of β-glucan (the primary PAMP for Dectin-1) on the surface of the fungal cells. Consistent with predictions, β-glucan surface exposure was significantly associated with mouse GI tract fitness across the entire range of *C. albicans* cell wall mutants and *Candida* species (Figure 5D). Overall these data indicate that the fungal cell wall may play an important role in the colonization of the mammalian GI tract, and that the level of β-glucan exposure on the cell surface correlates with a loss in competitive fitness of fungi in this host environment.

## Discussion

This study describes the generation of a carefully controlled set of isogenic cell wall mutants in *C. albicans* and provides a proof of concept of its usefulness in deciphering the role of the cell wall during *in vivo* host-microbe interactions. It also describes a number of optimized assays for precise quantitation of phenotypic parameters ranging from *in vitro* to *in vivo* fitness, which are expected to have broad applicability. In particular, we here show that by applying an unbiased, systematic, quantitative phenotypic characterization of a well-controlled set of *Candida* mutants and species, one can confidently rule in or rule out a number of hypotheses related to fungal colonization of the mammalian GI tract.

Most of the herein described mutants are disrupted in genes that had been knocked out from the *C. albicans* genome by other groups in the past. However, different genetic backgrounds have been used by different groups, some of which carrying auxotrophies or other mutations that might impact *in vivo* fitness (Bulawa et al., 1995; Hobson et al., 2004; Bates et al., 2005, 2006; Munro et al., 2005; Ben-Ami et al., 2011). Moreover, some of the laboratory strains previously used to disrupt some of these genes were later shown to carry large-scale chromosomal aberrations such as whole-chromosome aneuploidy and long-range loss-of-heterozygosity (Abbey et al., 2011). Such genomic alterations are likely to affect fungal fitness under a variety of conditions, and especially the highly stressful environment of the host (Pavelka et al., 2010; Selmecki et al., 2010). Moreover, following excision of the deletion cassette, no genetic marker was retained in the genome of our final mutants and their isogenicities were further verified by deep whole-genome sequencing. Finally, a long list of carefully quantified phenotypic information, including both *in vitro* and *in vivo* phenotypes, was systematically collected from the entire strain collection. For all these reasons, we believe that the herein described set of carefully constructed and deeply characterized *C. albicans* cell wall mutants will represent an important resource for the *Candida* research community at large, and in particular for investigations into the role of the fungal cell wall during host-microbe interactions.

The role of the fungal cell wall in host-microbe interactions has been extensively studied by several groups. *C. albicans* cell wall mutants have been employed to decipher the relative contribution of various cell wall components on immune responses and infection outcomes in both *in vitro* co-culture and systemic infection models. These studies have revealed the complexity and dynamics of the fungal cell wall and have shed light on how the host integrates information from multiple PAMPs through multiple PRRs to produce either protective or pathogenic immune responses. However, in spite of the mammalian GI tract being the main niche colonized by *Candida* species, little or nothing is known about the role played by the *Candida* cell wall during asymptomatic colonization of this niche. The most important finding in the present study is that the *Candida* cell wall indeed appears to play a role in determining the competitive fitness of these fungal species in the mouse GI tract.

However, GI tract fitness of the tested strains associated neither with the total amounts of individual cell wall components nor with *in vitro* fitness in a large number of tested environments. Two important lessons can be learned from these observations. First, predicting GI fitness from *in vitro* fitness in single environments is probably not possible because the GI tract is a very complex environment, which is very difficult to mimic *in vitro*. In fact, the GI tract is characterized by extremes of pH, gradients of nutrients and presence of a range of antimicrobial compounds like immunoglobulin A, bile salts and AMPs. The ability to survive and grow in face of any one of these individual challenges does not predict resistance to other stresses and hence does not predict overall fitness in the GI tract. The ideal colonizer most probably acts like a generalist that achieves suboptimal fitness across a wide range of individual environments rather than specializing in thriving in presence of any one given stress condition. Second, knowing the amount of chitin, mannans and glucans in the cell wall says little about how fit a *Candida* cell would be in the GI tract. The architecture of the cell wall, i.e., the spatial organization of various cell wall components relative to each other and to the cell surface, is far more important in determining how the host will recognize, interact with and respond to the microbe. In particular, it appears that the most critical determinant of GI tract fitness is the level of exposure of β-glucan on the cell surface, which associates with the strength of signaling through the Dectin-1 receptor on host cells. The dynamic nature of β-glucan masking and exposure was previously appreciated and shown to be linked to morphogenesis and to play an important role during systemic infection (Wheeler et al., 2008; Marakalala et al., 2013). Because of the profound negative impact of β-glucan exposure on the ability of *Candida* cells to colonize their main natural niche, i.e., the mammalian GI tract, we expect this phenomenon to be tightly regulated and this trait to be under strong evolutionary pressure.

## Author contributions

NP conceived, designed and supervised the study; XS, GL, AT, and GT performed microbiological, immunological, and mouse experiments; JL, KS, and FZ performed whole-genome sequencing experiments; MY, WL, MP and NP analyzed sequencing data; XS, GL, AT and NP performed statistical analyses.

## Funding

This work was supported by an A^*^STAR Investigatorship award (JCO/1437a00117), an NMRC Bedside-and-Bench grant (NMRC/BnB/0001b/2012) and by core funding from the Singapore Immunology Network to NP.

### Conflict of interest statement

The authors declare that the research was conducted in the absence of any commercial or financial relationships that could be construed as a potential conflict of interest.
