# Supplementary material for: β-glucan Exposure on the Fungal Cell Wall Tightly Correlates with Competitive Fitness of Candida Species in the Mouse Gastrointestinal Tract
Source: Front Cell Infect Microbiol. 2016 Dec 22;6:186. doi: 10.3389/fcimb.2016.00186 (PMC5177745; doi:10.3389/fcimb.2016.00186)
Supplement: Supplementary file 1 [file DataSheet1.docx]

### *Supplementary Materials*

**β-glucan exposure on the fungal cell wall critically determines competitive fitness of *Candida* species in the mouse gastrointestinal tract**

**XiaoHui Sem, Giang T. T. Le, Alrina S. M. Tan, Gloria Tso, Marina Yurieva, Webber W. P. Liao, Josephine Lum, Kandhadayar** **G. Srinivasan, Michael Poidinger, Francesca Zolezzi, Norman Pavelka***

***Correspondence:** [**norman_pavelka@immunol.a-star.edu.sg**](mailto:norman_pavelka@immunol.a-star.edu.sg)

#### 1 Supplementary Methods

#### Fungal strains and culture conditions

Stock cultures of all strains were preserved in 35% glycerol and maintained at −80°C. Cells were grown in Yeast Extract-Peptone-Dextrose (YPD) media (1% (w/v) yeast extract, 2% (w/v) peptone and 2% (w/v) D-glucose with 1.5% (w/v) agar for solid media only) at 37°C in a shaking incubator at 150-200 rpm unless otherwise specified. Hyphal growth was induced by either supplementing Dulbecco’s modified Eagle’s medium (DMEM) with 10% fetal bovine serum (FBS) (Gibco) or plating cells on Spider solid media (1% (w/v) nutrient broth, 0.4% (w/v) KPO_4_, 2% (w/v) D-glucose, 1.35% (w/v) agar).

**Construction of *C. albicans* strains**

The deletion constructs were generated using the *SAT1*-flipping strategy as described previously ([Sasse and Morschhauser, 2012](#_ENREF_13)). Briefly, plasmid pSFS2 containing the *SAT1* flipper cassette was used to disrupt the entire open reading frames of the *CHS3, OCH1, PMR1, MNT1, MNT2, MNN4* or *GSC1* genes of *C. albicans*. Flanking sequences 500-700 bp upstream or downstream of the targeted genes were first amplified from SC5314 genomic DNA by polymerase chain reaction (PCR) with primers listed in Table S2. The upstream and downstream PCR fragments of the respective target genes were then cloned sequentially as *KpnI-ApaI* and *SacII-SacI* fragments on both sides of the *SAT1* flipper cassette contained in plasmid pSFS2 to obtain the deletion constructs.

The resulting deletion constructs were digested with *KpnI* and *SacI* to excise the disruption cassette from the vector backbone and transformed into *SC5314* by electroporation ([Nguyen et al., 2009](#_ENREF_9)), with the exception of the double knockout mutant *mnt1/mnt1;mnt2/mnt2* , where *mnt1/mnt1* was used as the background strain for transformation. Transformants were subsequently selected on YPD plates containing 200 µg/ml nourseothricin (NAT, Jena Biosciences). Successful integration of the plasmid was confirmed by PCR with primers listed in Table S2. Positive transformants were then grown in yeast nitrogen base (YNB) media supplemented with maltose to induce the loss of the NAT selection marker and replica-plated onto YPD agar and YPD-NAT agar to identify heterozygous NAT-sensitive transformants ([Cottier et al., 2015b](#_ENREF_3)). Selected transformants were then used to obtain the homozygous mutants using the same process to disrupt the second allele of the target genes.

For the *C. albicans* SC5314-dTomato fluorescent strain, it was constructed by transforming the SC5314 reference strain ([Noble and Johnson, 2005](#_ENREF_10)) with the pENO1-dTom-NAT^R^ plasmid ([Gratacap et al., 2013](#_ENREF_5)). The dTomato gene in this plasmid is codon-optimized for *C. albicans* and constitutively expressed under the *ENO1* promoter. Transformation into the reference strain was carried out by electroporation as described above, using the *NotI* restriction enzyme for homologous recombination at the *ENO1* promoter. Successful integration of the plasmid was confirmed by PCR with primers listed in Table S2. Ten colonies were then selected and screened for red fluorescence by flow cytometry (561/615 nm, MACSQuant VYB, Miltenyi Biotec). Diploid DNA content was also verified by FACS analysis as described below. The brightest clone with proper integration and ploidy was then selected for use in subsequent experiments.

**Whole-genome sequencing**

#### A single colony was picked from an YPD-agar plate and grown overnight in 10 ml of YPD at 37°C under orbital shaking at 150 rpm. 1 ml of overnight culture was harvested by centrifugation at 3500 rpm for 5 minutes. Genomic DNA was extracted using the phenol-chloroform-isoamylalcohol method as previously described ([Rancati et al., 2008](#_ENREF_12)). The first set of libraries were prepared using Illumina TruSeq® DNA sample preparation kit version 2 (Low-Throughput protocol) according to manufacturer’s protocol except that size selection were done using E-Gel® SizeSelect™ 2% pre-cast agarose gels. Sequencing was performed using indexed paired-end (PE) sequencing runs of 2 × 51 bp on an Illumina HiSeq 2000 (HiSeq Control Software Version 2.2.58). The reconstructed *mnt1/mnt1*;*mnt2/mn2* strain (YNP422) and a new wild-type control strain (SC5314) were prepared using Illumina TruSeq® DNA PCR-Free library prep kit, enriching for DNA fragments of 550bp in length according to manufacturer’s instructions. The resultant libraries were sequenced using HiSeq 2500 on Rapid run mode to generate indexed PE reads of 2 × 251bp for SC5314 and 2 ×151bp for YNP422, respectively. Unless indicated otherwise, all sequencing data analysis was performed in CLC Genomics Workbench (version 8.0.3). Sequencing data are deposited in NCBI SRA database under accession number SRP056269.

#### For each sample, sequencing reads were first trimmed based on a quality score limit of 0.001 and minimal length of 15 bp. Trimmed paired-end reads were mapped to the *C. albicans* genome strain SC5314 (version A21-s02-m08-r09) using the following parameters: mismatch cost 2, insertion cost 3, deletion cost 3, length fraction 0.5, similarity fraction 0.8, and non-specific matching not permitted, i.e. reads that mapped with equal quality to more than one genomic location were ignored. A summary of the sequencing data is reported in Table S3. Small nucleotide changes, including single- and multiple-nucleotide variants and small insertions or deletions, were determined using the ‘Basic Variant Detection’ tool with following parameters: ploidy 2, minimum coverage and count 1, minimum variant frequency 1%, neighborhood radius 5, minimum central quality 20, minimum neighborhood quality 15. The ‘Amino Acid Changes’ tool was then used to annotate all non-synonymous changes in the called variants using the ‘12 Alternative Yeast Nuclear’ genetic code. Next, all variants were imported into the R environment, merged across samples and further filtered based on the following criteria. Variants present at allele frequency ≥1/3 in none or all samples were discarded. All resulting candidate mutations were then compared to a list of known polymorphisms in the SC5314 genome ([Jones et al., 2004](#_ENREF_7)) and the overlap was filtered out. Additional variants found in the two sequenced wild-type samples (WYU007 and WYY021) were also removed as background mutations. Finally, analysis was restricted to genomic positions with unambiguous nucleotides in the reference genome. Variants were considered significant if they were present with a minimum coverage of 10 and a minimum allele frequency of 1/3 in at least one mutant strain. Regardless of the previous filters, variants found across multiple independent strains at low frequency and/or coverage were also ignored.

#### Copy number variation (CNV) analysis was performed using CNV-seq software ([Xie and Tammi, 2009](#_ENREF_15)). A strain-specific window size was first selected by CNV-seq depending on the number of reads in the sample and the control strain, the size of the genome and the CNV detection threshold ratio r (−0.6 ≤ log_2_(r) ≤ 0.6, p-value < 0.001). The number of reads in each window was then divided by the number of reads in the control strain to normalize for differences in quantity of input DNA.

**Mouse experiments**

To assess the murine systemic virulence of each *C. albicans* strain and species, all strains were grown for 24 hours at 37°C in YPD media, harvested by centrifugation, washed twice with sterile PBS, and resuspended in sterile PBS. Fungal concentration was subsequently determined by the use of a hemocytometer. Female, 6-10 weeks old, C57BL/6 wild-type mice were housed in the Animal Biosafety Level 2 (ABSL2) cluster of the Biological Resource Centre (BRC) of A*STAR at a density of 4-5 mice per cage. Mice were infected intravenously via the lateral tail vein with each *C. albicans* or *Candida* spp. strain at a single dose of 5 x 10^5^ yeast cells in 50 μl of sterile PBS (n = 8 mice per group). Cages were regularly monitored over a 28-days observation period for dead or moribund mice, i.e. exhibiting severe lethargy, humped posture, lack of movement or >20% weight loss. Moribund mice were humanely euthanized using carbon dioxide-induced asphyxiation and their deaths recorded as occurring on the following day. Mice surviving the course of the experiment were humanely euthanized using similar methods on day 28. Mean survival for each group of mice were then calculated and plotted for comparison. All animal experiments and procedures were approved by the Institutional Animal Care and Use Committee (IACUC) of BRC in accordance with the guidelines of the Agri-Food and Veterinary Authority (AVA) and the National Advisory Committee for Laboratory Animal Research (NACLAR) of Singapore.

**Competition experiments**

To establish gastrointestinal (GI) tract colonisation of *C. albicans* strains and other *Candida spp.*, mice were first depleted of their indigenous GI bacterial flora using methodologies as modified and adapted from ([Koh et al., 2008](#_ENREF_8)). Briefly, mice were administered 2 mg streptomycin/ml (Sigma) and 1500 U penicillin G/ml (Sigma) added to their sterile drinking water for 3-4 days. Similarly-treated sterile drinking water was changed every 3-4 days. To assess the GI tract fitness of each *C. albicans* and *Candida* species relative to the reference SC5314-dTomato strain, all strains were grown for 24 hrs at 37^o^C in YPD media, harvested by centrifugation, washed twice with sterile PBS, and resuspended in sterile PBS. Fungal concentration was subsequently determined by the use of a hemocytometer. For *C. albicans* strains, an aliquot of the PBS resuspension was treated with a mixture of 0.1 M Tris-hydrochloride pH 8.1 (Promega), 0.5 M magnesium sulphate (Sigma), 12 mM dithiothreitol (Merck-Calbiochem) and 1 mg/ml zymolase (USBiological) for 1.5 hrs in a 37^o^C water bath prior to counting to dissolve cell clumps. Antibiotic-treated mice were then singly housed in separate cages and infected by a single oral gavage of 10^7^ yeast cells in 100 μl of sterile PBS in a 1:1 mixture of the test strain (*C. albicans* mutant or *Candida* species) and the reference strain (SC5314-dTomato). At specific time points, a fresh stool pellet was collected from each mouse and mechanically homogenized in sterile PBS. Each stool homogenate was then filtered through a 40 μm-cell strainer nylon filter (BD), 10-fold serially diluted in sterile PBS and plated evenly on YPD agar plates containing penicillin-streptomycin. The initial inoculum was also serially diluted in a similar way and plated on YPD-agar plates to determine the relative frequency of the two strains prior to initiation of the competition experiment. All agar plates were incubated at 37^o^C for 24 hrs. To measure the relative frequencies of each strain, ≥200 red fluorescent and non-fluorescent colony-forming units (CFUs) were counted under a fluorescence stereomicroscope (Olympus). Relative ratios of the number of non-fluorescent colonies vs. the total number of colonies were then calculated and the relative fitness of the different strains/species against SC5314-dTomato were then determined by using a linear regression model with the formula

log_2_[*R*(*t*)*/R*(*t*_0_)] *= sγ*_R_(*t−t_0_*),

where *R*(*i*) represents the ratio between the test strain and the reference strain at time *i*; *s* is the selection coefficient; *γ*_R_ is the growth rate of the reference strain expressed as cell divisions per hour; *t* represents the time points in hours and *t*_0_ the initial time point. Because the *in vivo* growth rate of *C. albicans* is unknown and difficult to determine, we obtained the value of the expression *sγ_R_* as the slope of the linear regression and used it as an indirect measure of *in vivo* relative fitness.

*In vitro* competition experiments were performed essentially in the same way, except that 1:1 mixtures of the reference fluorescent SC5314-dTomato strain against each of the competing strains/species were first obtained by mixing 6.25×10^5^ cells of each strain in 50 ml of fresh YPD medium. The competing mixture was then serially passaged at a concentration of 1.25×10^6^ cells over 4 days at 37°C in a shaking incubator at 150 rpm. Total number of cells were then counted using a haemocytometer every 10-12 hrs, diluted and plated onto YPD agar plates containing penicillin-streptomycin before colony-counting under the fluorescence stereomicroscope. Because *γ_R_* could be easily determined for the SC5314-dTomato reference strain grown alone in YPD, we obtained *s* as the slope of the linear regression and used this as a direct measure of *in vitro* competitive fitness.

***Candida*-macrophage co-cultures**

J774.1 murine macrophages (ATCC) were cultured in Dulbecco’s modified Eagle’s medium (DMEM) supplemented with 10% (v/v) fetal calf serum (FBS) and 0.5% (v/v) penicillin-streptomycin (10,000U/ml) (Invitrogen/Gibco), in tissue culture plates (Corning) at 37°C and 5% CO_2_. The macrophage cell line J774.1 was plated at a density of 0.5×10^6^ cells in 12-well plates (Corning) for 24 hrs. Wild-type and mutant *C. albicans* strains from overnight cultures were washed twice with sterile PBS, counted and co-cultured with 1×10^6^ macrophages at multiplicities of infection (MOIs) of 1:200, 1:400, 1:800 and 1:1,600 *C. albicans*/macrophages. The cultures were then incubated on ice for 30 min before transferring to 37°C and 5% CO_2_. In these low-MOI settings, no free-floating *Candida* cell was visible under a microscope 3 hours after initiation of co-culture, indicating ~100% phagocytosis efficiency for all tested strains. Fungal colonies became visible 24 hours after co-culture initiation, indicating successful escape from the macrophages. At the same time, due again to the low MOIs used, no massive macrophage cell death was observed at this time point. As a loading control, *Candida* cells were plated under identical growth conditions but without the presence of macrophages and at cell densities as low as to being equivalent to an MOI of 1:12,800. The percentage of fungal cells able to escape from macrophages was determined by the following method. The average number of visible colonies from 3 technical replicates was first fitted against the MOI (or MOI equivalent in the case of the loading control) according to a linear model without intercept. The ratio between the so-obtained slopes was then used as an estimate of the relative proportion of plated fungal cells that were able to form colonies. Reported values are from a minimum of 3 independent experiments.

**Quantitative resistance assays**

The effects of weak organic acids (WOAs) on *C. albicans* strains and *Candida* spp. were determined *in vitro* via turbidimetric growth assays as described previously ([Cottier et al., 2015a](#_ENREF_2)) with some minor modifications. Strains were inoculated into 96-deep-well blocks at a starting OD_600_ of 0.1 in a final volume of 1.7 ml fresh YPD media with different concentrations of WOAs, incubated at 37°C under orbital shaking at 200 rpm. The pH was adjusted to 5.5 by the use of either 5M hydrochloric acid or 10M sodium hydroxide solution. Growth data was obtained by regularly sampling the cultures with a Freedom EVO 150 automated liquid-handling robot (Tecan) and measuring the OD_600_ of each well with a SpectraMax Plus384 absorbance microplate reader (Molecular Devices). Changes in OD over time were then fitted against a dose response curve and used to determine the half maximal inhibitory concentration (IC_50_) of the tested WOA. All assays were repeated with four independent biological replicates.

NO sensitivity assay was performed following a previous protocol ([Chiranand et al., 2008](#_ENREF_1)) with some minor modifications. Overnight cultures were diluted to an OD_600_ of 0.1 and grown at 37°C for 2 hrs in YPD media pH 7.4 (pH adjusted by HCl). Cultures were then divided into 5 ml aliquots. Freshly prepared DPTA NONOate (a polyamine NO donor with a half-life of 3 hrs at 37°C pH 7.0 to 7.4) solution were dissolved in the same medium and added to each aliquot to final concentrations of 0, 0.1, 0.2, 0.3, 0.5, 1 and 2 mM. Cells were incubated at 37°C for 3.5 hrs and cell density was compared by OD_600_ measurements. Data was normalized by subtracting the OD_600_ at 0 hr from the OD_600_ at 3.5 hrs and setting the cell density measurement in the 0mM DPTA NONOate condition as equal to 100. Remaining cell density measurements (at OD_600_), recorded over the range of DPTA NONOate concentrations for each strain, were then expressed as a fraction of the maximum for that strain recorded during that particular experiment. Four independent experiments were performed on each set of strains.

**High-throughput phenotypic profiling**

*C. albicans* strains and *Candida* spp. were first revitalized by streaking out glycerol stocks onto fresh YPD agar and sub-cultured into YPD media overnight at 37°C. OD_600_ measurements of the overnight cultures were taken the next day and normalized to a final OD of 2. Normalized cultures were centrifuged at 3,500 rpm for 5 mins and washed once with PBS. 100 μl from each culture was then aliquoted into individual wells on the first column of a 96-well plate. With the help of the Freedom EVO 150 liquid-handling robot, serial dilutions of the cultures were performed and 3 μl of each serial dilution were spotted onto YPD agar omnitrays containing various cell wall perturbing agents and grown under conditions listed in Table S5. Plates were scanned after 24-48h on a desktop scanner and images were processed via a fully automated script written in the R language ([R Core Team, 2014](#_ENREF_11)). Briefly, the ‘gitter’ package was used for spot detection and quantification ([Wagih and Parts, 2014](#_ENREF_14)). We then used the ‘nls’ function to perform a non-linear curve fitting of the spot intensities as a function of the serial dilutions according to a logistic function, and obtained the dilution required to reach 50% of the maximum spot intensity of each strain. Relative growth scores were then calculated first by normalizing the dilution value against the dilution value of the WT control strain spotted on the same plate, followed by division of this normalized dilution value against the corresponding normalized dilution obtained from a YPD control plate spotted in parallel to the plate containing the tested stress condition. Each tested condition had a minimum of three biological replicates. The R script is available from authors upon request.

**Quantification of β-1,3-glucan exposure**

Quantification of β-1,3-glucan exposure on the surface of *C. albicans* was done as previously published (Wartenberg et al., 2014), with some modifications. 200 µl of logarithmically-growing *C. albicans* suspension was pelleted in a 96-well plate by centrifugation at 3,500 rpm for 5 minutes. Cells were fixed with 1% formaldehyde, washed twice with PBS and blocked with 1% BSA for 1 hour at room temperature with gentle shaking, before incubation with an anti-β-1,3-glucan monoclonal antibody (mouse IgG, Biosupplies; 1:600 dilution in 1% BSA) overnight at 4°C with gentle shaking, followed by three washing steps in PBS, incubation with an Alexa Fluor 488-coupled anti-mouse IgG (Molecular Probes; 1:300 dilution) for 1 hour at room temperature with gentle shaking and three final washing steps in PBS. Relative fluorescent intensities were then calculated by taking the ratio of the tested sample stained with/without secondary Alexa Fluor 488-coupled anti-mouse IgG antibody.

**2 Supplementary Figures**

**
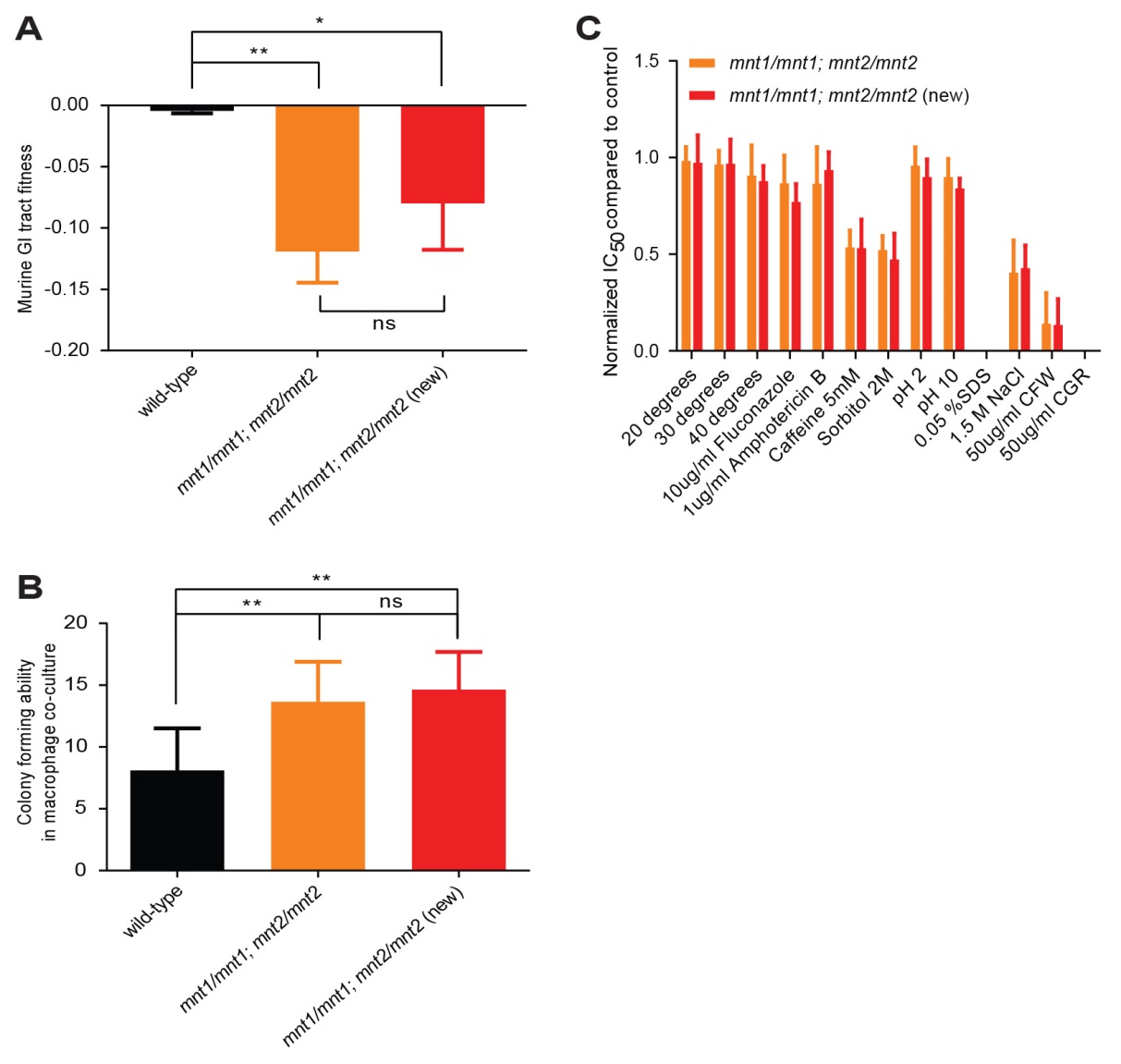
**

**Supplementary Figure 1:** No significant difference in murine GI tract fitness and phenotypic analyses observed in reconstructed *mnt1/mnt1*;*mnt2/mnt2* (new) mutant as compared to the original *mnt1/mnt1*;*mnt2/mnt2* mutant.

**(A-B)** Murine GI tract fitness and the ability to escape from macrophages after phagocytosis were reassessed in the *mnt1/mnt1*;*mnt2/mnt2* (new) mutant and found not to be significantly different from the original *mnt1/mnt1*;*mnt2/mnt2* mutant*.* Asterisks shown represent significant two-tailed *p* values obtained from unpaired t-tests with Welch’s correction between wild-type *C. albicans* and the indicated strain (* < 0.05; ** < 0.01). **(C)** Summary of quantitative phenotypic analyses performed on the *mnt1/mnt1*;*mnt2/mnt2* (new) mutant in comparison to the original *mnt1/mnt1*;*mnt2/mnt2* mutant. Experimental conditions are as summarized in Table S5. SDS = sodium dodecyl sulfate; CFW = Calcofluor White; CGR = Congo Red.

**
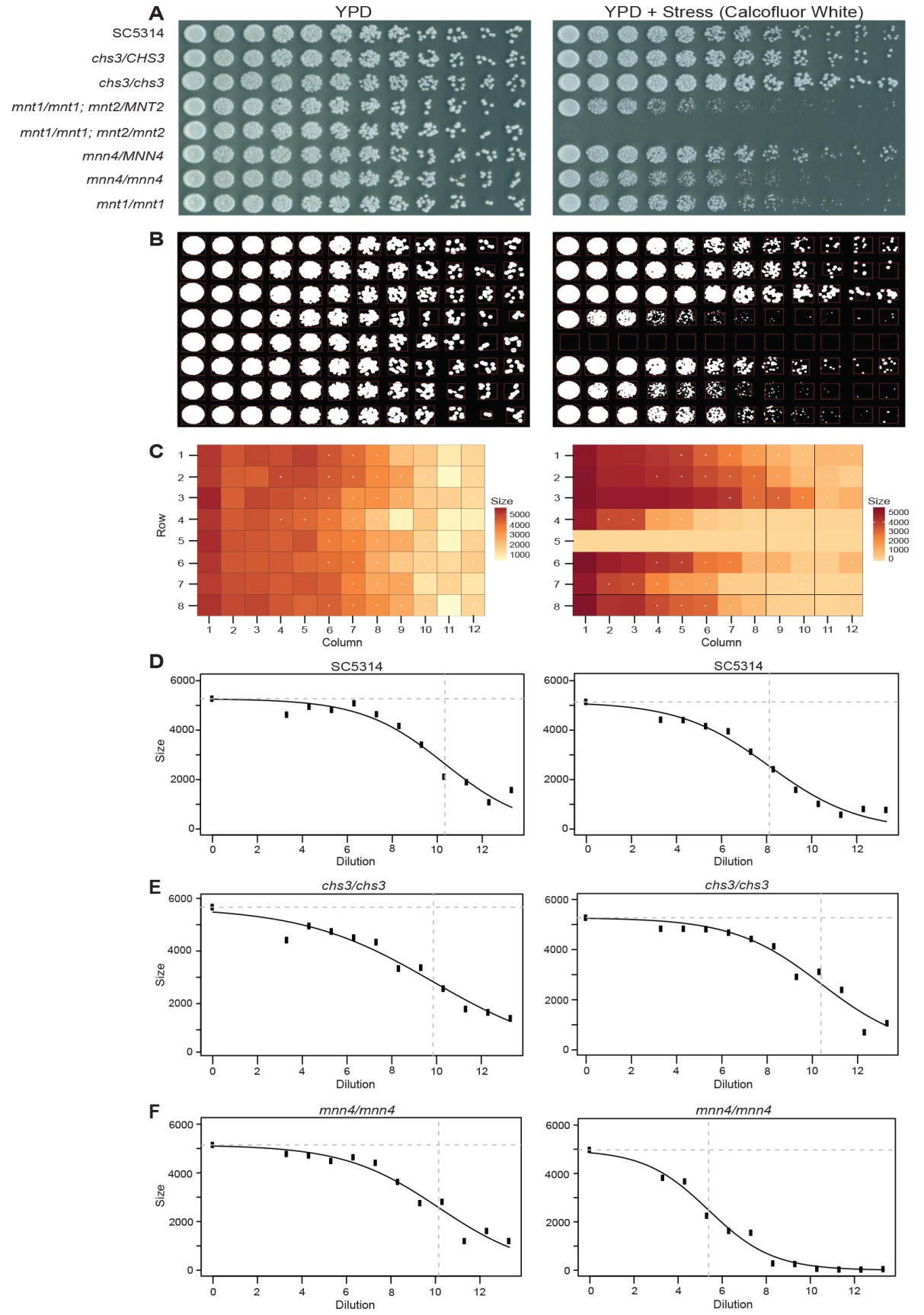
**

**Supplementary Figure 2:** High-throughput phenotypic profiling using a customized R script for automated spot detection and intensity measurements acquired from agar spotting assays

**(A)** Representative growth data of wild-type SC5314 *C. albicans* and selected isogenic cell wall mutants as spotted onto YPD agar omnitrays with or without Calcofluor White. **(B)** Automated spot detection of the various *C. albicans* strains under the listed condition using a customized R script. **(C)** Quantified spot intensities as acquired by the R script are represented as heatmaps. **(D-F)** Dose-response curves of selected *C. albicans* strains as acquired by intensity measurements followed by non-linear curve fitting across the range of serial dilutions and determination of relative growth scores. Horizontal dashed line: fitted upper asymptote; vertical dashed line: fitted value corresponding to the dilution required to reach 50% of asymptotic spot intensity.


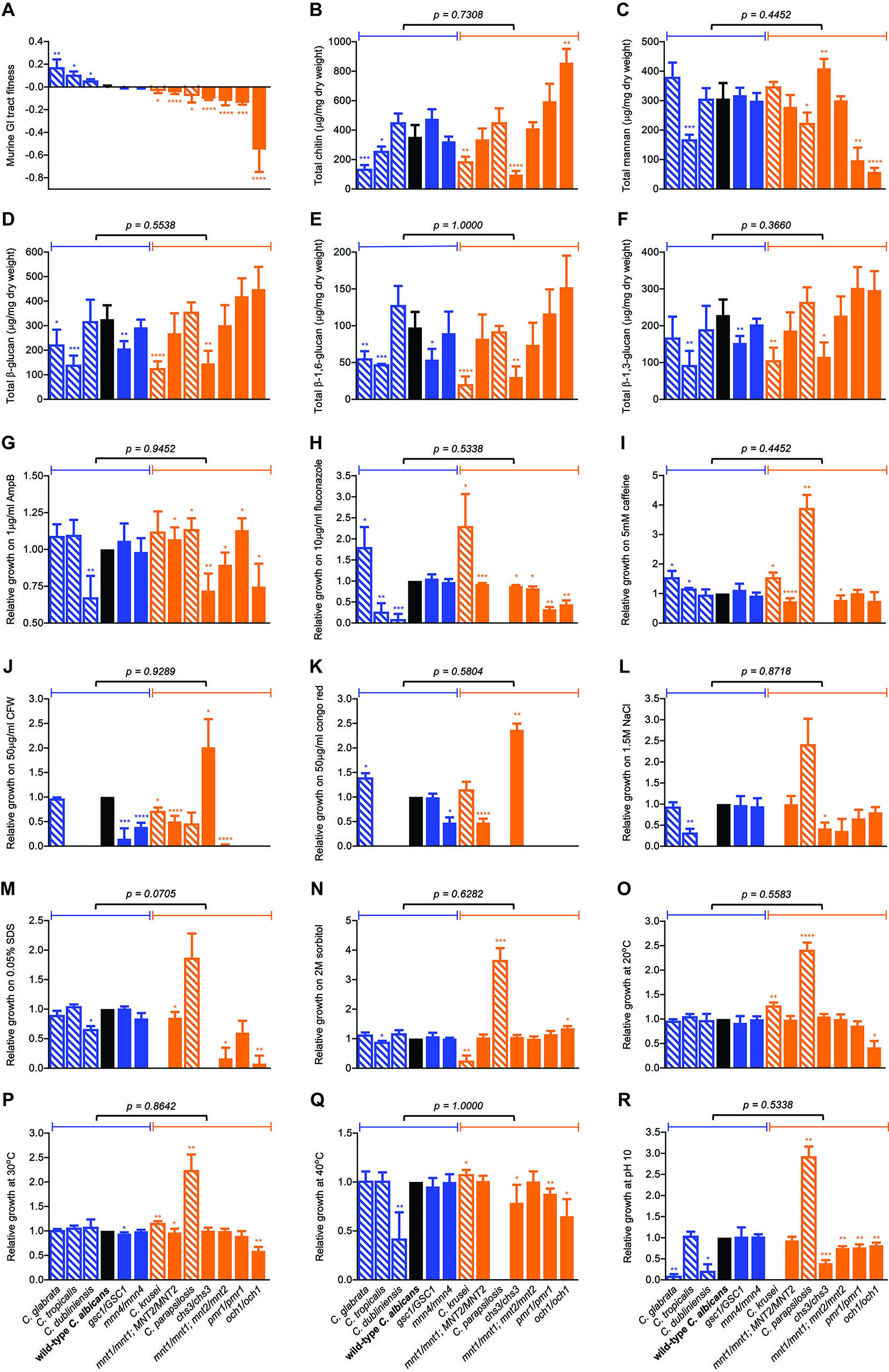


**Supplementary Figure 3:** Factors not associated with murine GI tract fitness

**(A)** Wild-type *C. albicans* (black), isogenic *C. albicans* cell wall mutants (solid blue and orange) and *Candida spp.* strains (shaded blue and orange) were assessed for their GI tract fitness in a colonisation assay as competing strains with SC5314-dTomato (same data as shown in Fig. 2F and 3F). Strains are ranked in decreasing order of murine GI tract fitness and classified into two groups: fitter than or as fit as wild-type *C. albicans* (solid or shaded blue); less fit than wild-type *C. albicans* (solid or shaded orange). **(B-D)** Cell wall polysaccharide composition analysis data of *C. albicans* cell wall mutants and of *Candida* species shown in Figure 2G-I and 3G-1 were combined here for ease of comparison. Using more specific procedures, all strains were also assessed for their **(E)** total β-1,6-glucan and **(F)** total β-1,3-glucan levels. Using the high-throughput phenotypic profiling method, strains were then further assessed for their **(G)** relative growth on 1 μg/ml amphothericin B (AmpB), **(H)** 10 μg/ml fluconazole, **(I)** 5 mM caffeine, **(J)** 50 μg/ml Calcofluor white (CFW); **(K)** 50 μg/ml Congo red, **(L)** 1.5 M NaCl, **(M)** 0.05% SDS; **(N)** 2 M sorbitol, **(O)** relative growth at 20°C, **(P)** 30°C, **(Q)** 40°C and **(R)** pH 10. All *p* values shown are obtained from non-parametric Mann-Whitney tests, comparing the following two unpaired groups: wild-type *C. albicans*, isogenic *C. albicans* cell wall mutants and *Candida spp.* strains fitter than or as fit as wild-type *C. albicans* (blue bracket); isogenic *C. albicans* cell wall mutants and *Candida spp.* strains less fit than wild-type *C. albicans* (orange bracket). Asterisks shown represent significant two-tailed *p* values obtained from unpaired t-tests with Welch's correction between wild-type *C. albicans* and the indicated strain (* < 0.05; ** < 0.01; *** < 0.001; ****< 0.0001).

**
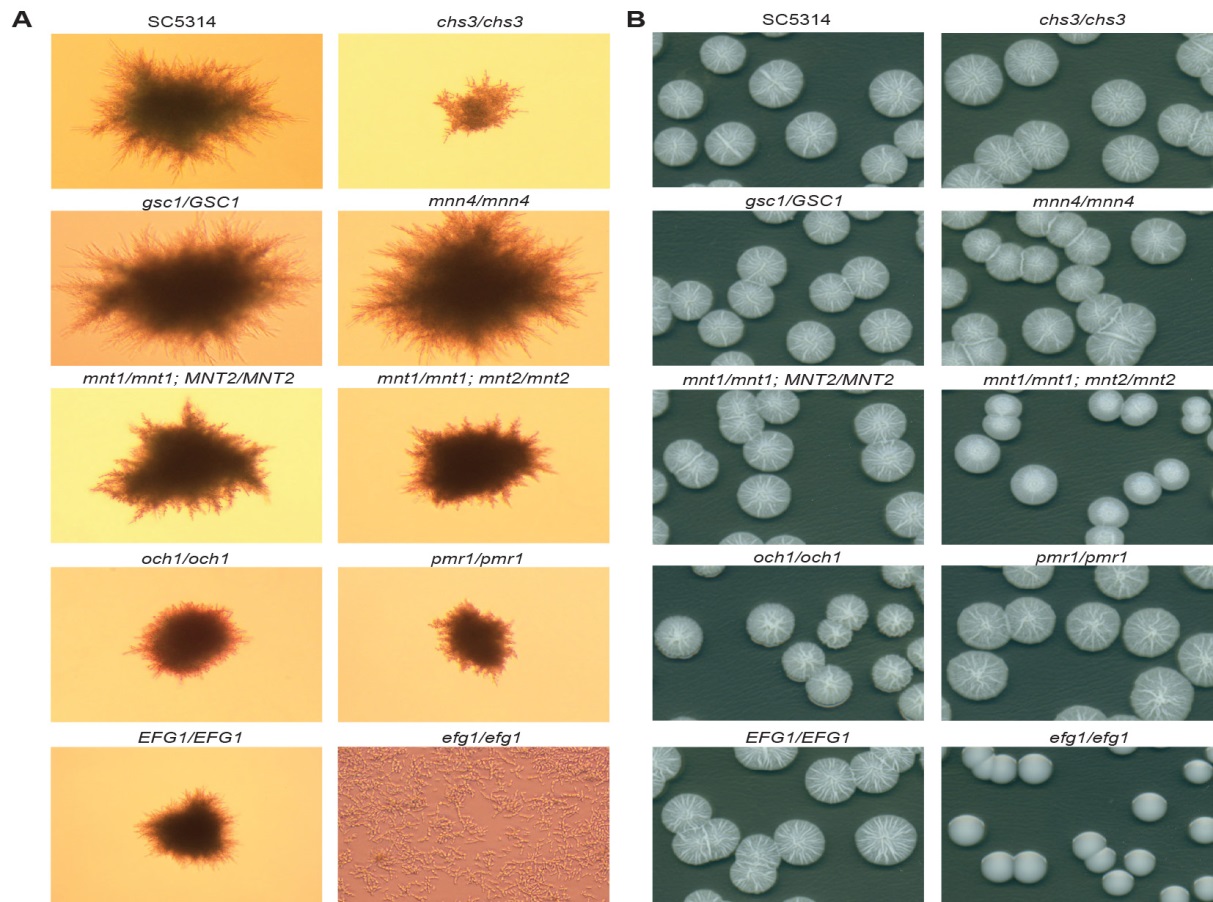
**

**Supplementary Figure 4:** No hyphae formation defects in cell wall mutants

Colony morphology of the different *C. albicans* cell wall mutants after growth in **(A)** Dulbecco’s modified Eagle’s medium (DMEM) supplemented with 10% (v/v) fetal calf serum (FBS) at 37°C and 5% CO_2_ for 24 hrs and **(B)** Spider medium at 37°C over 4 days. Photomicrographs of DMEM cultures were acquired at a magnification of 4×. Spider plate images were acquired on a desktop scanner.

**3 Supplementary Tables**

### Supplementary Table 1: *Candida* strains and *Candida* species used in this study

| **Strain ID** | **Alias** | **Karyotype** | **Genotype** | **Reference** |
| --- | --- | --- | --- | --- |
| YNP19 | SC5314 | 2N | Wild type | ([Noble and Johnson, 2005](#_ENREF_10)) |
| YNP53 | *chs3/CHS3* | 2N | *CHS3/Δchs3::FRT* | This study |
| YNP52 | *chs3/chs3* | 2N | *Δchs3/Δchs3::FRT* | This study |
| YNP43 | *och1/OCH1* | 2N | *OCH1/Δoch1::FRT* | This study |
| YNP44 | *och1/och1* | 2N | *Δoch1/Δoch1::FRT* | This study |
| YNP45 | *pmr1/PMR1* | 2N | *PMR1/Δpmr1::FRT* | This study |
| YNP46 | *pmr1/pmr1* | 2N | *Δpmr1/Δpmr1::FRT* | This study |
| YNP47 | *mnt1/MNT1* | 2N | *MNT1/Δmnt1::FRT* | This study |
| YNP48 | *mnt1/mnt1* | 2N | *Δmnt1/Δmnt1::FRT* | This study |
| YNP51 | *mnt1/mnt1; mnt2/MNT2* | 2N | *MNT2/Δmnt2::FRT;Δmnt1/Δmnt1::FRT* | This study |
| YNP50 | *mnt1/mnt1; mnt2/mnt2* | 2N | *Δmnt2/Δmnt2::FRT;Δmnt1/Δmnt1::FRT* | This study |
| YNP422 | *mnt1/mnt1; mnt2/mnt2* (new) | 2N | *Δmnt2/Δmnt2::FRT;Δmnt1/Δmnt1::FRT* | This study |
| YNP55 | *mnn4/MNN4* | 2N | *MNN4/Δmnn4::FRT* | This study |
| YNP54 | *mnn4/mnn4* | 2N | *Δmnn4/Δmnn4::FRT* | This study |
| YNP49 | *gsc1/GSC1* | 2N | *GSC1/Δgsc1::FRT* | This study |
| YNP73 | *SC5314*- dTomato | 2N | Wild type *pENO1-dTom-NAT^r^* | This study |
| YNP3 | GA1 | 2N | *C. parapsilosis* | ([Gacser et al., 2007](#_ENREF_4)) |
| YNP69 | ATCC 2001 | 2N | *C. glabrata* | ATCC |
| YNP92 | ATCC 14243 | 2N | *C. krusei* | ATCC |
| YNP93 | ATCC 13803 | 2N | *C. tropicalis* | ATCC |
| YNP94 | ATCC MYA-646 | 2N | *C. dubliniensis* | ATCC |
| YNP423 | *EFG1/EFG1* | 2N | SN152 | ([Homann et al., 2009](#_ENREF_6)) |
| YNP424 | *efg1/efg1* | 2N | efg1::hisG/efg1::hisG-URA3-hisG | ([Homann et al., 2009](#_ENREF_6)) |

**Supplementary Table 2:** Primers used in this study

| **Primer** | **Sequence (5’ → 3’)^1^** |
| --- | --- |
| CHS3upF1 | TGTGGTAATAAGAAGATAATGAGG |
| CHS3upF | TTTTTT**GGTACC**TAATGTTGTAAAAAGACCTGCTG (KpnI) |
| CHS3upR | TTTTTT**GGGCCC**TTCCTAAACCTTCACCAGATTC (ApaI) |
| CHS3doF | TTTTTT**CCGCGG**ATCTTCTTCAGGGTCCAGTTG (SacII) |
| CHS3doR | TTTTTT**GAGCTC**TTAATTTTGGGACCCTTGAGTA (SacI) |
| CHS3doR1 | GAATGCTAAATGTTCAGTATCCTA |
| CHS3rtF | ATTTTCGGTGGGGTTACTTG |
| CHS3rtR | AGGTAACCCGAAAATAACTGC |
| OCH1upF1 | TTTTTCCATTTTTGTTACTTTTGA |
| OCH1upF | TTTTTT**GGTACC**ATTGTATTATTAGTTGTCATCC (KpnI) |
| OCH1upR | TTTTTT**GGGCCC**TTATAGTAAAGGTCTCGTGTC (ApaI) |
| OCH1doF | TTTTTT**CCGCGG**ATATGTTTCTGGGAAGTTGG (SacII) |
| OCH1doR | TTTTTT**GAGCTC**AGGATTAAAGACCGCTGTG (SacI) |
| OCH1doR1 | CTCAATATCGAGAGCGTGTGC |
| OCH1rtF | CAACACTTCGACAACAACTTTCA |
| OCH1rtR | CTTAGCAATCAATTCACGCAAC |
| PMR1upF1 | AGTGGATGAAGAGGACAAGGTG |
| PMR1upF | TTTTTT**GGTACC**AAGGGATAGACGAAACAAACG (KpnI) |
| PMR1upR | TTTTTT**GGGCCC**CTTGAGTGATGGGGTGTTTG (ApaI) |
| PMR1doF | TTTTTT**CCGCGG**TGTATAGCTTGCAGGAGGGAG (SacII) |
| PMR1doR | TTTTTT**GAGCTC**CCAACCAAATGAAATAGTCGG (SacI) |
| PMR1doR1 | AGCATGGTGTCGGTCTCTAAAT |
| PMR1rtF | GTGACAGAATCCCCGCAGAC |
| PMR1rtR | CCTTTGAAGTCGGCTGTCCA |
| MNT1upF1 | TACGCGTTACACCAAGATACAA |
| MNT1upF | TTTTTT**GGTACC**GCAGTTGTCACTACCGCCTC (KpnI) |
| MNT1upR | TTTTTT**GGGCCC**CCCAAATCTAATCAAACGAGC (ApaI) |
| MNT1doF | TTTTTT**CCGCGG**TCAGGACAGGAAACGAAGATAG (SacII) |
| MNT1doR | TTTTTT**GAGCTC**TCTACTACGCACCTCCTCCAT (SacI) |
| MNT1doR1 | TGAAAATATTAGGCATCCACAAG |
| MNT1rtF | CACTTTGGCCCGTAACTCTG |
| MNT1rtR | CGTCAGAAACCCAATCCATC |
| MNT2upF1 | AATTTCTTTTTGGCTTCTGGA |
| MNT2upF | TTTTTT**GGTACC**CTTTTCCACCAAAACACCAAC (KpnI) |
| MNT2upR | TTTTTT**GGGCCC**ATGGTGGTTAAGATGATTGCC (ApaI) |
| MNT2doF | TTTTTT**CCGCGG**TTTACAAAAACCTAAAGAATGGG (SacII) |
| MNT2doR | TTTTTT**GAGCTC**GAGGTCGAGTATTATATGGAGAAG (SacI) |
| MNT2doR1 | TGGTATGGTGGGAATGAAAG |
| MNT2rtF | TTATGATTGGTATTGGAGAGTTG |
| MNT2rtR | GATATTTTTCCCATTCTTTAGGT |
| MNT2rtF1 | ACCACCACAATCACCTTCATC |
| MNT2rtR1 | TTTTCAAATCAATCCAATCAGG |
| MNN4upF1 | TATTCGTTCCGTCTCATCATTC |
| MNN4upF | TTTTTT**GGTACC**ACAACAACAGCAACGATTCAAC (KpnI) |
| MNN4upR | TTTTTT**GGGCCC**TGTGTTTGTTGATTGAGGGAAG (ApaI) |
| MNN4doF | TTTTTT**CCGCGG**AAATTATCGCTTTTACATCAGG (SacII) |
| MNN4doR | TTTTTT**GAGCTC** ATCCTTTGTTTGCTTTTTATTG (SacI) |
| MNN4doR1 | TCACCTGCTACTTCTATTGTTGG |
| MNN4rtF | AACAAGAAGAGAAAGCCAATGA |
| MNN4rtR | ATTATTGGCAAGGTATGATTCTG |
| GSC1upF1 | GATCTTGGTGTTTTGTTCAGTTC |
| GSC1upF | TTTTTT**GGTACC**GTGCGTGTGAGTTGGTAAAAG (KpnI) |
| GSC1upR | TTTTTT**GGGCCC**GTATATCAGTTGGGTGTTGGTTT (ApaI) |
| GSC1doF | TTTTTT**CCGCGG**TCCAACCAAGAAATGTCAGTAAT (SacII) |
| GSC1doR | TTTTTT**GAGCTC**AAATCTTTTATGGTCGTGTTCG (SacI) |
| GSC1doR1 | TGTTTACTCTTTGTATGGGTTGG |
| GSC1rtF | TGGATTTTTCATTGCTGTGG |
| GSC1rtR | TGAACGTGATCAATGGCTAAC |
| FLP rtF | AGGTGCTTGTTCGTCAGTTTG |
| FLP rtR | TGTGGCTATTTCCCTTATCTGC |
| SAT1 rtF | AATTTCGGTGATCCCTGAGC |
| SAT1 rtR | TGTTTCGTTCGAGACTTGAGG |
| pENO1-dTom FW | TCCTTGGCTGGCACTGAACTCG |
| pENO1-dTom REV | AAGGTCTACCTTCACCTTCACC |

^1^) Sequence highlighted in underline and bold corresponds to the unique recognition sequence of the restriction enzymes (as stated in parentheses) used in this study.

**Supplementary Table 3:** Mapping and coverage summary of whole genome sequencing of mutant strains.

| **Sample ID** | **Strain ID** | **Strain genotype** | **Sequencing strategy^1^** | **Total # of reads** | **# of mapped reads** | **% of mapped reads** | **Average sequencing depth** | **% of genome covered by ≥ 1 read** | **% of genome covered by ≥ 10 reads** |
| --- | --- | --- | --- | --- | --- | --- | --- | --- | --- |
| WYY014 | YNP44 | *och1Δ/och1Δ* | A | 22,262,764 | 21,406,397 | 96.15% | 69.89 | 99.50% | 99.23% |
| WYY015 | YNP46 | *pmr1Δ/pmr1Δ* | A | 22,615,902 | 21,780,433 | 96.31% | 71.14 | 99.48% | 99.20% |
| WYY016 | YNP48 | *mnt1Δ/mnt1Δ* | A | 22,654,954 | 21,803,775 | 96.24% | 71.21 | 99.48% | 99.20% |
| WYY017 | YNP49 | *gsc1Δ/GSC1Δ* | A | 19,886,750 | 19,184,237 | 96.47% | 62.64 | 99.45% | 99.16% |
| WYY018 | YNP50 | *mnt1Δ/mnt1Δ*;*mnt2Δ/mnt2Δ* | A | 24,686,574 | 23,800,014 | 96.41% | 77.69 | 99.45% | 99.20% |
| WYY019 | YNP52 | *chs3Δ/chs3Δ* | A | 20,639,190 | 19,893,528 | 96.39% | 65.00 | 99.42% | 99.11% |
| WYY020 | YNP54 | *mnn4Δ/mnn4Δ* | A | 21,927,738 | 21,130,680 | 96.37% | 69.05 | 99.48% | 99.20% |
| WYY021 | SC5314 | wild-type | A | 23,277,560 | 22,385,952 | 96.17% | 73.16 | 99.44% | 99.17% |
| WYY041 | YNP422 | *mnt1Δ/mnt1Δ*;*mnt2Δ/mnt2Δ* (new) | B | 13,838,756 | 13,332,581 | 96.34% | 91.52 | 99.79% | 99.65% |
| WYU007 | SC5314 | wild-type | C | 17,122,190 | 16,326,374 | 95.35% | 117.38 | 99.83% | 99.72% |

**^1^**) A = Libraries were prepared using Illumina TruSeq® DNA sample preparation kit version 2 (Low-Throughput protocol). Sequencing was performed using indexed paired-end (PE) sequencing runs of 2 × 51 bp on an Illumina HiSeq 2000. B = Libraries were prepared using Illumina TruSeq DNA PCR-free kit. Sequencing was performed using indexed PE sequencing runs of 2 × 151 bp on an Illumina HiSeq 2500 (Rapid run mode). C = Libraries prepared as in B. Sequencing was performed using indexed PE sequencing runs of 2 × 251 bp on an Illumina HiSeq 2500 (Rapid run mode)

**Supplementary Table 4:** Genome sequence differences between *C. albicans* cell wall mutants

| **Sample ID** | **Strain genotype** | **Chromosome** | **Position** | **Nucleotide change** | **Change type** | **Locus** | **Amino acid change** | **Zygosity** |
| --- | --- | --- | --- | --- | --- | --- | --- | --- |
| WYY014 | *och1Δ/och1Δ* | Chr2 | 393435 | C>T | non-synonymous | *ARO3* | Ser219Leu | heterozygous |
|  |  | Chr3 | 1385404 | A>G | non-synonymous | *REV3* | Arg1587Gly | heterozygous |
|  |  | Chr3 | 1386941 | A>G | intergenic | - | - | heterozygous |
| WYY015 | *pmr1Δ/pmr1Δ* | Chr3 | 456430 | T>A | non-synonymous | *STE50* | Tyr89Asn | heterozygous |
|  |  | Chr3 | 465196 | T>A | non-synonymous | *ERG6* | Asn350Ile | heterozygous |
|  |  | Chr3 | 496300^496301 | ->A | intergenic | - | - | heterozygous |
|  |  | Chr7 | 47961 | C>T | non-synonymous | *CA5609* | Val10Ile | heterozygous |
|  |  | Chr7 | 48045 | A>- | intergenic | - | - | heterozygous |
|  |  | Chr7 | 51345 | T>C | intergenic | - | - | heterozygous |
|  |  | ChrR | 1787873 | A>G | intergenic | - | - | heterozygous |
| WYY016 | *mnt1Δ/mnt1Δ* | Chr1 | 10402 | C>G | intergenic | - | - | heterozygous |
|  |  | Chr3 | 398842 | A>G | intergenic | - | - | heterozygous |
|  |  | Chr5 | 867674 | T>G | intergenic | - | - | homozygous |
| WYY017 | *gsc1Δ/GSC1Δ* | Chr1 | 10402 | C>G | intergenic | - | - | heterozygous |
| WYY018 | *mnt1Δ/mnt1Δ*; *mnt2Δ/mnt2Δ* | Chr1 | 10402 | C>G | intergenic | - | - | heterozygous |
|  |  | Chr1 | 809983 | G>A | synonymous | *BNI4* | - | heterozygous |
|  |  | Chr3 | 398842 | A>G | intergenic | - | - | heterozygous |
|  |  | Chr3 | 405142 | T>C | non-synonymous | *BGL98* | Ser864Pro | homozygous |
| WYY041 | *mnt1Δ/mnt1Δ*; *mnt2Δ/mnt2Δ* (new) | Chr1 | 10402 | C>G | intergenic | - | - | heterozygous |
|  |  | Chr3 | 398842 | A>G | intergenic | - | - | heterozygous |
|  |  | Chr5 | 659988 | T>G | non-synonymous | *YPT35* | Gln69Pro | heterozygous |
|  |  | Chr5 | 822342 | C>T | intergenic | - | - | heterozygous |
|  |  | Chr6 | 911078 | G>- | intergenic | - | - | heterozygous |
|  |  | ChrR | 510829 | C>T | synonymous | *PGA23* | - | heterozygous |
| WYY019 | *chs3Δ/chs3Δ* | Chr1 | 2399836 | G>T | non-synonymous | *CA3338* | Ser460Tyr | heterozygous |
|  |  | Chr1 | 2860483 | A>G | intergenic | - | - | heterozygous |
| WYY020 | *mnn4Δ/mnn4Δ* | No mutations found | | | | | | |

**Supplementary Table 5:** Cell wall perturbing agents and other stresses used in the phenotypic profiling

| **Condition^1^** | **Description of perturbation** |
| --- | --- |
| 37 °C | Control condition |
| 20 °C | Hypothermia |
| 30 °C | Suboptimal temperature |
| 40 °C | Hyperthermia |
| pH 2 | Acid stress |
| pH 10 | Alkaline stress |
| Sodium chloride (NaCl) 1.5 M | High osmolarity |
| Sodium dodecyl sulphate (SDS) 0.05% | Cell wall stress |
| Sorbitol 2 M | Cell wall stress |
| Caffeine 5 mM | Cell wall stress |
| Congo red 50 μg/ml | Cell wall stress |
| Calcofluor White (50 μg/ml) | Cell wall stress |
| Fluconazole (10 μg/ml) | Antifungal drug |
| Amphotericin B (1 μg/ml) | Antifungal drug |
| Ox bile (1%) | Bile stress |
| Bile salt (1 g/L) | Bile stress |

^1^) The base medium for all plates was YPD.

#### 4 Supplementary References

Chiranand, W., Mcleod, I., Zhou, H., Lynn, J.J., Vega, L.A., Myers, H., Yates, J.R., 3rd, Lorenz, M.C., and Gustin, M.C. (2008). CTA4 transcription factor mediates induction of nitrosative stress response in Candida albicans. *Eukaryot Cell* 7**,** 268-278.

Cottier, F., Tan, A.S., Chen, J., Lum, J., Zolezzi, F., Poidinger, M., and Pavelka, N. (2015a). The transcriptional stress response of Candida albicans to weak organic acids. *G3 (Bethesda)* 5**,** 497-505.

Cottier, F., Tan, A.S., Xu, X., Wang, Y., and Pavelka, N. (2015b). MIG1 Regulates Resistance of Candida albicans against the Fungistatic Effect of Weak Organic Acids. *Eukaryot Cell* 14**,** 1054-1061.

Gacser, A., Trofa, D., Schafer, W., and Nosanchuk, J.D. (2007). Targeted gene deletion in Candida parapsilosis demonstrates the role of secreted lipase in virulence. *J Clin Invest* 117**,** 3049-3058.

Gratacap, R.L., Rawls, J.F., and Wheeler, R.T. (2013). Mucosal candidiasis elicits NF-kappaB activation, proinflammatory gene expression and localized neutrophilia in zebrafish. *Dis Model Mech* 6**,** 1260-1270.

Homann, O.R., Dea, J., Noble, S.M., and Johnson, A.D. (2009). A phenotypic profile of the Candida albicans regulatory network. *PLoS Genet* 5**,** e1000783.

Jones, T., Federspiel, N.A., Chibana, H., Dungan, J., Kalman, S., Magee, B.B., Newport, G., Thorstenson, Y.R., Agabian, N., Magee, P.T., Davis, R.W., and Scherer, S. (2004). The diploid genome sequence of Candida albicans. *Proc Natl Acad Sci U S A* 101**,** 7329-7334.

Koh, A.Y., Kohler, J.R., Coggshall, K.T., Van Rooijen, N., and Pier, G.B. (2008). Mucosal damage and neutropenia are required for Candida albicans dissemination. *PLoS Pathog* 4**,** e35.

Nguyen, L.N., Trofa, D., and Nosanchuk, J.D. (2009). Fatty acid synthase impacts the pathobiology of Candida parapsilosis in vitro and during mammalian infection. *PLoS One* 4**,** e8421.

Noble, S.M., and Johnson, A.D. (2005). Strains and strategies for large-scale gene deletion studies of the diploid human fungal pathogen Candida albicans. *Eukaryot Cell* 4**,** 298-309.

R Core Team (2014). "R: A Language and Environment for Statistical Computing". (Vienna, Austria: R Foundation for Statistical Computing).

Rancati, G., Pavelka, N., Fleharty, B., Noll, A., Trimble, R., Walton, K., Perera, A., Staehling-Hampton, K., Seidel, C.W., and Li, R. (2008). Aneuploidy underlies rapid adaptive evolution of yeast cells deprived of a conserved cytokinesis motor. *Cell* 135**,** 879-893.

Sasse, C., and Morschhauser, J. (2012). Gene deletion in Candida albicans wild-type strains using the SAT1-flipping strategy. *Methods Mol Biol* 845**,** 3-17.

Wagih, O., and Parts, L. (2014). gitter: a robust and accurate method for quantification of colony sizes from plate images. *G3 (Bethesda)* 4**,** 547-552.

Xie, C., and Tammi, M.T. (2009). CNV-seq, a new method to detect copy number variation using high-throughput sequencing. *BMC Bioinformatics* 10**,** 80.
